# Steering light by a sub-wavelength metallic grating from transformation optics

**DOI:** 10.1038/srep12219

**Published:** 2015-07-17

**Authors:** Yadong Xu, Yangyang Fu, Huanyang Chen

**Affiliations:** 1 College of Physics, Optoelectronics and Energy & Collaborative Innovation Center of Suzhou Nano Science and Technology, Soochow University, No. 1 Shizi Street, Suzhou 215006, China

## Abstract

Transformation optics has shown great ability in designing devices with novel functionalities, such as invisibility cloaking. A recent work shows that it can also be used to design metasurfaces which usually come from the concept of phase discontinuities. However, metasurfaces from transformation optics have very complicated material parameters. Here in this work, we propose a practical design, a sub-wavelength metallic grating with discrete and gradient index materials. Such a design not only inherits some functionalities of metasurfaces from phase discontinuities, but also shows richer physics. Our work will also provide a guidance to recent activities of acoustic metasurfaces, especially for those made of extremely anisotropic metamaterials.

It is a significant topic to manipulate the propagation of light in controllable ways. Such a design is available by employing artificial metamaterials^1^,^2^,^3^, where arbitrary electromagnetic (EM) parameters could be obtained by local resonances of microstructures in a sub-wavelength scale. Using them, some extraordinary physical phenomena have been demonstrated, such as invisible cloaking^4^,^5^, negative refraction^6^,^7^, field rotators^8^, polarization manipulation^9^,^10^, etc. As a category of metamaterials, an ultrathin planar form, called metasurfaces, recently have drawn much attention, as they exhibit unusual optical phenomena^11^,^12^,^13^,^14^ and potential for applications^15^,^16^,^17^,^18^,^19^. In particular, by introducing phase discontinuities along an interface, anomalous reflection or refraction of light could be observed^11^,^12^,^13^, which is concluded by the generalized Snell’s law. For a well designed metasurface, the phase discontinuities will bring about a new critical angle, which can be deduced from the generalized Snell’s law. When the incident angle is beyond such a critical angle, the incident wave will be converted into surface wave propagating along the interface. This feature has been employed to design a functional metasurface, which can build a bridge between the propagating wave and the surface wave^14^. Besides, another set of generalized Snell’s law from view of transformation optics, was proposed to control EM wave propagation for all polarizations of incident light^20^, while the required EM parameters of the designed metasurface are quite complicated. To implement them more easily, a reduced version which works only for a transverse magnetic(TM) wave, was also displayed by using extremely anisotropic metamaterials^20^, e.g., *μ*_*z*_ = *ε*_*x*_ = *n*(*x*) and *ε*_*y*_ = −∞.

In order to realize such an anisotropic material property, in this work, we propose a metallic grating structure made of a silver screen perforated by periodic slits. To manipulate the direction of the outgoing wave, a discrete phase shift within a period fully covering 2π is introduced along the outgoing interface of the grating structure by filling the slits with different media. Based on numerical simulations, by exploring the situations of full incident angles, we find that the generalized Snell’s law could not always work effectively, but needs to be further amended. For incident angles within the critical angle, the anomalous refraction still can be described accurately by the generalized Snell’s law. While for incident angles beyond the critical angle, the incident wave will not just be converted into surface wave, but will mainly transmit through the metallic grating structure by matching the required tangential wave vector from a higher propagating order. Such unusual transmitted wave can be predicted by a new law of refraction involving diffraction effect of super lattices, which has been successfully used to explain the effect of extraordinary beam-steering in recent work of acoustic metasurfaces^21^.

## Results

### Model and Theory

**Figure 1:**
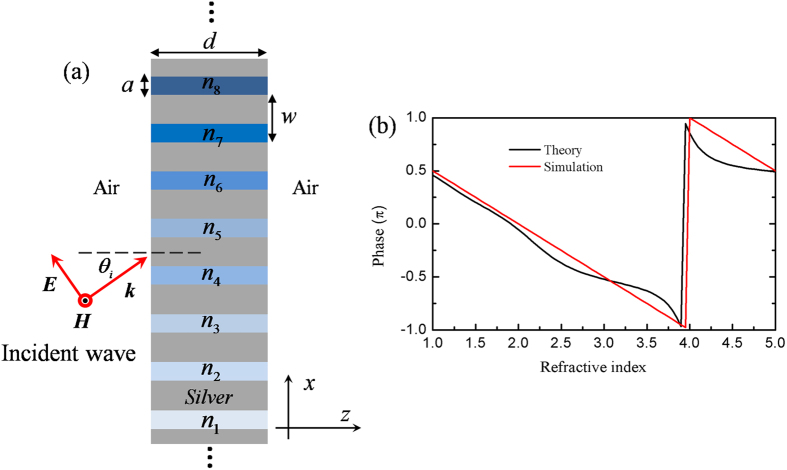
Periodical metallic grating with gradient indexes. (**a**) The schematic of the metallic grating, where only one supercell is displayed. (**b**) The relationship of relative phase shift and refractive index. The red line is calculated approximately by *φ *= *βd*, while the black curve is the numerical result for a normally incident TM wave. In simulations, the parameters *w*, *a* and *d* are set as 1 *μm*, 0.75 *μm* and 2 *μm*, respectively. The impedances of all filled media are matched to the air.

The schematic of such a metallic grating is illustrated in Fig. 1(a), where the metallic grating is composed of periodically repeated supercells. Every supercell is made of a silver screen corrugated by slits of a width *a.* The separation of two adjacent slits is *w* and the thickness of silver screen is *d*. In our design, each supercell contains 8 slits. Thus the period of each supercell is *p* = 8*w.* A TM wave with magnetic field along *y* direction is incident from left to the grating, and the working wavelength is set as *λ* = 8 *μm*. If all the silts are only filled with air, for a sub-wavelength metallic grating (*i.e.*, *w* << λ), only zero-order diffraction is involved for the outgoing wave. To steer its propagating direction at will, by following the concept of metasurfaces, we introduce a phase shift *ϕ*(*x*) along the interface between the metallic grating and the medium in right. In order to produce a continuous and smooth wave-front of outgoing wave, the variation range in a supercell should fully cover 2*π*. Therefore, the 8 slits in each supercell are filled with materials of gradient refractive indexes and marked by *n*_1_, *n*_2_, …, *n*_8_ from bottom to top side in Fig. 1(a). Moreover, for two adjacent slits in each supercell numbered as *l* and *l* + 1, the phase difference along the right interface is Δ*ϕ* *=* *π*/4.

Before giving out concretely what media are filled in each supercell, let us inspect briefly EM wave inside metallic silts. In fact, such silts can be viewed as metallic waveguide arrays. As the widths of each tiny “waveguide” is very small, only fundamental modes could be supported in each slit, and they are independent of the incidence angle. The wave number *β* can be ascertained by the following equation^22^,
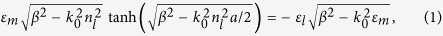
where *k*_0_ = 2*π/λ* is the wave number in air, 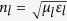 is the refractive index of the medium filled in the *l*-slit of each supercell, with *ε*_*l*_ and *μ*_*l*_ indicating, respectively, its relative permittivity and permeability, and *ε*_*m*_ is the relative permittivity of silver. From equation (1), we know that the wave number *β* is only dependent of the refractive index *n*_*l*_ for a fixed width *a* of slits. Moreover, for *λ* = 8 *μm*, silver can be treated as perfect electric conductors (PECs), as a result the guided mode in each slit is a transverse electric and magnetic (TEM) mode. Based on this, the wave number can be approximately given by *β* = *n*_*l*_*k*_0_. Accordingly, after the incident EM wave tunnels through the metallic waveguide arrays, the total phase of EM wave at the right interface could be written as,
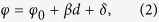
where *φ*_0_ is the initial phase at the left, *βd* is the retardant phase stemming from the medium inside each slit, and the last term *δ* is the phase resulted from the multiple reflections between the left and right interfaces. From equation (2), we find that the phase retardation *φ* of EM wave at the right interface may be adjusted by changing the filled medium. Figure 1(b) shows the relationship between relative retardant phase and refractive index, in which the red curve is the analytical result calculated by *φ* *= βd*, while the black curve is the numerical result when the TM wave is normally incident to the metallic grating. In simulations, we set *d* *=* *λ*/4, *w* = *λ*/8 and *a = *0.75*w*. Moreover, the corresponding refractive index in the *l*-th silt of each supercell is given by *n*_*l*_ = 1 + (*l *− 1)*λ*/*md*, where *m* is the total number of slits in a supercell (here *m* = 8) and *l* denotes the order of the corresponding slit. In order to diminish the reflection of incident wave from the metallic grating structure and the multiple scattering between the left and right interfaces, the impedances of the filled media are matched to air, that is *ε*_*l*_ = *μ*_*l*_ = *n*_*l*_. It is clearly seen that as the refractive index is changed from 1 to 5, the variation of retardant phase *φ* can cover the full range of 2*π*. Comparing the analytical result with the numerical one, we find that both results agree very well. Such a grating structure could be regarded as an implementation of the reduced version of metasurface from transformation optics^20^.

Now let’s explore what will happen for outgoing wave when the TM wave transmits through the designed metallic grating. For simplicity, we suppose that both sides of such a metallic grating are filled with air. The outgoing wave is called refractive wave with refractive angle *θ*_*t*_ determining the outgoing direction. As we have introduced phase shifts along the interface in the metallic grating, which is analogous to the phase discontinuities of metasurfaces, therefore, at first glance, its propagating direction should be governed by the generalized Snell’s law^11^,
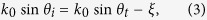
where *ξ* = *dϕ*(*x*)/*dx* = Δ*ϕ*/Δ*x* is an additional momentum induced essentially by the phase shifts along the right interface. For the current design, it has Δ*ϕ* = *π*/4 and Δ*x* = *w* = *p*/8, leading to 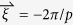 (here the “−” sign means that the direction of vector is along −*x* direction). From the view of metasurfaces, there is a critical angle *θ*_c_, which is deduced from the generalized Snell’s law with *θ*_*t*_ = 90°, *i.e., arcsin*(1 − *ξ*/*k*_0_). When the incident angle *θ*_*i*_ is beyond the critical angle *θ*_c_, that is *θ*_*i*_ > *θ*_c_, in the design of metasurfaces, the refractive wave will disappear and the incident wave will be converted into surface wave propagating along the interface. But in the proposed structure, the fact is not that simple and straightforward. In particular, when *θ*_*i*_ > *θ*_c_, the incident wave will not just be converted into surface wave, but will mainly pass through the metallic grating with a new propagating direction. Therefore, for the case of *θ*_*i*_ > *θ*_c_, the generalized Snell’s law of equation (3) will be out of order, but needs to be further amended. Based on analysis and massive numerical simulations, we find that the refractive wave obeys a new law of refraction,
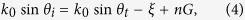
where *n* is an integer, and *G* = 2*π*/*p* is the reciprocal lattice of supercells. It is noted that although both vectors of *ξ* and *G* have the same value in quantities, yet they have different physical meanings. The former is introduced by the phase shifts along the interface, whereas the latter is caused by the periodic structure of supercells. Such a new law of refraction has been employed to predict successfully anomalous beam-steering effect in recent work of acoustic metasurfaces^21^, where it is corresponding to *n* = 3 for equation (4) when *θ*_*i*_ > *θ*_c_.

### Analysis from iso-frequency contours

**Figure 2:**
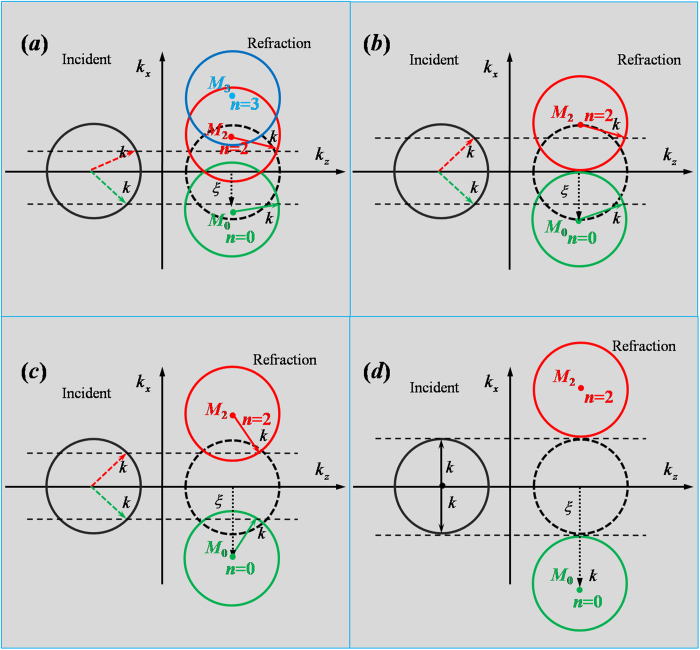
Iso-frequency contours for different additional momentums *ξ*. (**a**–**d**) are corresponding to four situations that 0 < *ξ* *<* *k*_0_, *ξ* =* k*_0_, *k*_0_ <*ξ* < 2*k*_0_ and 2*k*_0_ ≤ *ξ*, respectively. In all cases, the solid and dashed black circles represent the iso-frequency contours of air corresponding to incident side and refracted side, respectively. Both black circles, with their centers located at *k*_*x*_ axis, have the same radius. The green circles indicate the iso-frequency contours for the cases of introducing phase shift *ξ* with *n *= 0 in equation (4), *i.e.,* moving the dashed black one down with *ξ*, while the red and blue circles are iso-frequency contours for the cases of introducing reciprocal lattice corresponding to *n *= 2 and *n *= 3 in equation (4), respectively. When the incident wave is illuminating to the metallic grating from air, the green (or red) dashed arrows in (**a**–**d**) denote the incident directions. After refraction, the directions of refractive wave are marked by the green (or red) solid arrows. Two paralleled dashed lines indicate the tangential momentum conservation.

In order to illustrate the directions of refractive wave for different incident angles, we employ the iso-frequency contours to intuitively uncover the inherent physics. Four situations sorted by different additional momentum *ξ* are considered, as shown in Fig. 2. Figure 2(a–d) are corresponding to the cases of 0 < *ξ* < *k*_0_, *ξ* = *k*_0_, *k*_0_ < *ξ* < 2*k*_0_ and *ξ* ≥ 2*k*_0_, respectively. In each case, if there is no phase shift along the interface, the iso-frequency contours in free space are merely indicated by two identical black circles, where the solid one is for the incident wave, while the dashed one is for the refracted wave. Once the phase shifts are introduced, then at the refracted side, the dashed black circle will be shifted down with a displacement of *ξ*, as marked by the green circle. Such a physical picture follows the generalized Snell’s law in equation (3), or the new law in equation (4) with *n* = 0. However, when *θ*_*i*_ > *θ*_c_, due to the periodic structure of supercells, the grating diffractions should be taken into consideration, the refractive wave will abide by the new law of refraction, which can be depicted by the iso-frequency contours in red or blue. For example, when *n* = 2, the green circle will shift up to the red one with a displacement 2*G*. In the following, we will study the refractive phenomena of transmitted wave for different *ξ*.

For the case of 0 < *ξ* < *k*_0_ as shown in Fig. 2(a), the green, red and blue circles denotes the iso-frequency contours for the refractive wave corresponding to *n *= 0, *n *= 2 and *n *= 3 in equation (4), respectively. As described by equation (3), we know that the critical angle is *θ*_c_ = *arcsin*(1 − *ξ*/*k*_0_) > 0. When −90° < *θ*_*i*_ ≤ *θ*_c_, the refractive angle *θ*_t_ is predicted by the generalized Snell’s law in equation (3). An example is shown by green arrows in iso-frequency circles. When *θ*_c_ < *θ*_*i*_ < 90°, the incident wave cannot couple to the green circle. If we examine this situation from the view of metasurfaces, it should be converted into surface wave. But in current metallic grating structure, however, the refractive wave couples to a new propagating order *n *= 2, *i.e.,* the red circle in Fig. 2(a), and its direction is indicated by the red solid arrow by matching the tangential momentum. It is noted that because the reciprocal lattice vector G is not quite large, when the incident angle is large enough, the refractive wave can couple not only to the propagating order *n *= 2, but also possibly to a higher propagating order *n *= 3, *i.e.,* the blue circle in Fig. 2(a). Under such circumstances, it is very difficult from the iso-frequency contours to learn which propagating order the refractive wave will take.

For the case of *ξ* = *k*_0_ in Fig. 2(b), the corresponding critical angle is *θ*_*c*_ = 0. Similarly, when −90° < *θ*_*i*_ ≤ 0, the refractive angle *θ*_*t*_ obeys the generalized Snell’s law, as shown by the green arrows in Fig. 2(b). While for 0 < *θ*_*i*_ < 90°, the refractive direction can be predicted by a new law of refraction in equation (4) with *n *= 2 (see red arrows in Fig. 2(b)). Specially, for a normal incident wave, the refractive angle is 90°, which means that the refractive wave propagates along the interface of metallic grating and air.

For the case of *k*_0 _< *ξ *< 2*k*_0_, the corresponding critical angle is *θ*_*c*_ < 0°. For −90° < *θ*_*i*_ < *θ*_*c*_, the refractive direction is still following the generalized Snell’s law, which is displayed by the green arrows in Fig. 2(c), while for −*θ*_*c*_ < *θ*_*i*_ < 90°, the refractive beam is determined by the new law of refraction with *n *= 2, which is described by red arrows in Fig. 2(c). However, for *θ*_*c*_ < *θ*_*i*_ < −*θ*_*c*_, among which the incident wave could not couple to such new propagating orders, *i.e.,* red and green circles. It is also quite difficult for refractive wave to couple to the propagating mode *n *= 1, as shown by the dashed black circle. As a result, the incident wave will be partially converted into the surface wave, and partially be reflected by the grating structure, with extremely weak transmitted wave whose refractive angle is predicted by the black dashed circle (*i.e.,* it follows Snell’s law *θ*_*t*_ = *θ*_*i*_). This interesting feature is magnified for the case of *ξ* ≥ 2*k*_0_, as shown by Fig. 2(d), in which the incident wave could not couple to the green and red circles regardless of any incident angle.

### Proofs by numerical simulations

To illustrate the above idea, numerical simulations are performed by using COMSOL Multiphysics to show the performance of steering wave or beam by such a metallic grating with phase shifts along the interface. Four examples with *ξ *= 0.8*k*_0_, *ξ *= *k*_0_, *ξ *= 1.2*k*_0_ and *ξ *= 2*k*_0_ are demonstrated, and the corresponding periodicities of supercell *p* are 10 *μm*, 8 *μm*, 6.7 *μm* and, 4 *μm*, respectively. In all simulations, the working wavelength is *λ* = 8 *μm* and the thickness of grating is 2 *μm*.

#### (a) Metallic grating with *ξ* = 0.8*k*_0_

**Figure 3:**
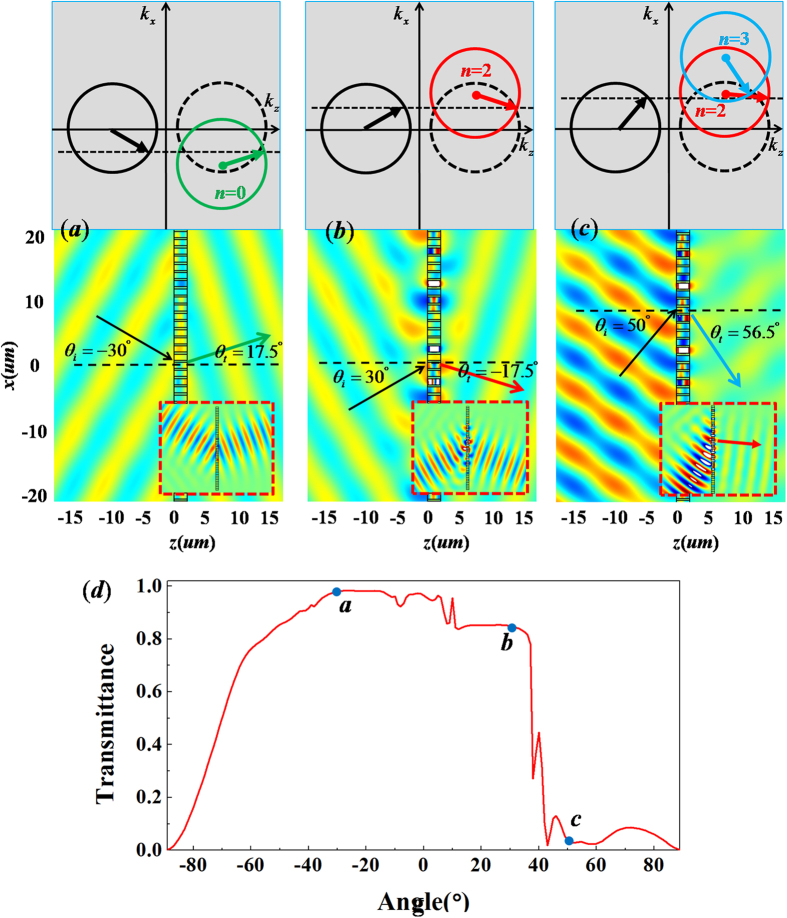
The case of metallic grating with *ξ* = 0.8*k*_0_. (**a**–**c**) are simulated magnetic field patterns for incident wave with different angles with *θ*_*i*_ = −30°, 30° and 50° respectively. The upper parts are the corresponding iso-frequency contours, while the corresponding patterns of plane wave incident on metallic grating are placed in the nether parts, where the patterns for Gaussian beams bumping on the metallic grating with 6 supercells are inserted in bottom, which are marked by the red dashed frames. (**d**) is relationship between transmittance and incident angle. The points a, b and c in (**d**) denote the corresponding transmittance for the above cases of (**a**–**c**), respectively.

Based on above analysis, we know that the critical angle is *θ*_*c*_ = 11.55° for *ξ* = 0.8*k*_0_. For *θ*_*i*_ = −30° < *θ*_*c*_, the refractive angle is *θ*_*t*_ = 17.5°, as predicted by the generalized Snell’s law. Such a result also is described by the iso-frequency contours in Fig. 3(a). To verify this calculated refractive angle, we carry out numerical simulation for the case of *θ*_*i*_ = −30°. As shown by the field pattern in Fig. 3(a), the refractive angle is indeed about 17.5°, which is consistent with theoretical result. To make it more straightforward, we suggest a more practical case, where a Gaussian beam with *θ*_*i*_ = −30° bumps on the metallic grating with 6 supercells (see the insertion in Fig. 3(a)), and an apparently negative refraction with *θ*_*t*_ = 17.5° is displayed. When the incident angle is beyond the critical angle, as we predict previously, the incident wave is not just converted into surface wave, but mainly transmits through the metallic grating with a new propagating order *n *= 2. In such case, the refractive wave will follow the new law of refraction, as described by the iso-frequency contour in Fig. 3(b) and the corresponding refractive angle is expressed as, 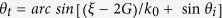. Suppose that the incident angle is *θ*_*i*_ = 30° > *θ*_*c*_, the calculated refractive angle is about −17.5°, which is well demonstrated by the numerical results in Fig. 3(b) where the refractive angle is observed as −17.5° for both plane wave and Gaussian beam. In addition, we can find obvious evidence of Spoof Surface Plasmon (SSP) localized at both interfaces of metallic grating. If the incident angle is large enough, the incident wave may couple to a higher propagating order, *e.g.,* the *n *= 3 order as marked by the blue circle in iso-frequency contours of Fig. 3(c). The corresponding refractive angle is predicted by 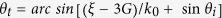. On this occasion, the incident wave may transmit through the metallic grating with the two propagating orders, *i.e., n *= 2, 3. In order to make clear this confusing problem, the numerical simulation for plane wave with *θ*_*i*_ = 50° is shown in Fig. 3(c), where the transmitted wave is very weak, but the refractive angle is *θ*_*t*_ = 56.5°, consistent with the theoretical result obtained by 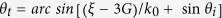. However, from the simulation of Gaussian beam(see the insertion in Fig. 3(c)), the refractive angle *θ*_*t *_= 2° is almost consistent with the result predicted by 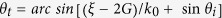. The relationship between the wave transmittance and incident angle is revealed in Fig. 3(d). We can observe that when the incident angle is within the critical angle, the transmittance is very high, because the transmitted wave is propagating wave by following the generalized Snell’s law. When the incident angle is beyond the critical angle (*i.e.,* 17.5° < *θ*_*i*_ < 37° in Fig. 3(d)), as the incident wave can couple to the new propagating order of *n* = 2, with a little part confined in the metallic grating as SPP. As a result, the transmittance will be reduced slightly. However, if the incident angle is large enough (*i.e., θ*_*t*_ > 37°, which is the critical angle for coupling to a higher propagating order *n* = 3 exactly), most part of the wave might be converted into SSP, while the refractive wave can take both propagating orders of *n *= 2 and *n *= 3, with low transmittance.

#### (b) Metallic grating with *ξ* = *k*_0_

**Figure 4:**
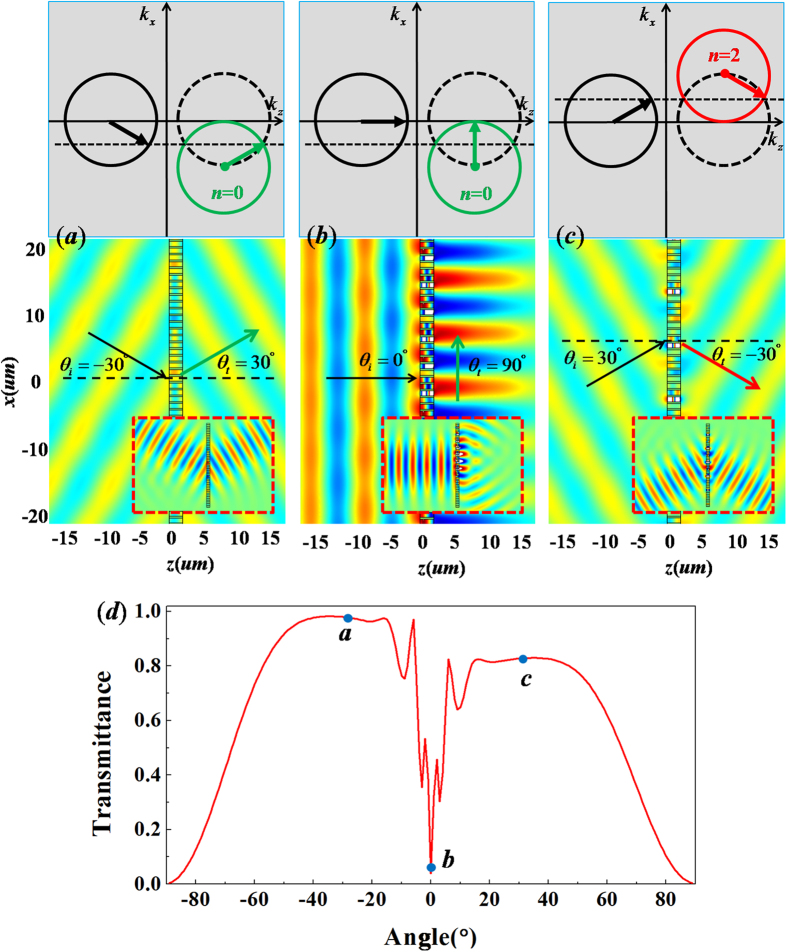
The case of metallic grating with *ξ* = *k*_0_. (**a**–**c**) are simulated magnetic field patterns for incident wave with different angles with *θ*_*i*_ = −30°, 0° and 30°, respectively. The upper parts are the corresponding iso-frequency contours, while the corresponding patterns of plane wave incident on metallic grating are placed in the nether parts, where the patterns for Gaussian beams bumping on the metallic grating with 6 supercells are inserted in bottom, which are marked by the red dashed frames. (**d**) is relationship between transmittance and incident angle. The points a, b and c in (**d**) denote the corresponding transmittance for the above cases of (**a**–**c**), respectively.

In this case, the critical angle is *θ*_*c*_ = 0°. Figure 4(a) numerically shows the result that a plane wave with *θ*_*i*_ = −30° < *θ*_*c*_ is incident on the metallic grating and we can find that the incident plane wave could pass through the grating with a refractive angle *θ*_*t*_ = 30°, which is consistent with the calculated refractive angle obtained by the generalized Snell’s law (see the iso-frequency contours in Fig. 4(a)). The similar result for *θ*_*i*_ = −30° is simulated by a Gaussian beam bumping on the metallic grating with 6 supercells (see the insertion in Fig. 4(a)). When a plane wave is incident with *θ*_*i*_ = *θ*_*c*_ = 0°, the refractive angle should be *θ*_*t*_ = 90°, which is also displayed by the iso-frequency contours in Fig. 4(b) and this implies that the outgoing plane wave can propagate along the surface of metallic grating, which is demonstrated by the numerical simulation in Fig. 4(b). The corresponding simulation result of a Gaussian beam with *θ*_*i*_ = 0° is also inserted in Fig. 4(b), where we can see that the transmitted wave propagates along the surface of metallic grating structure composed of 6 supercells. However, when the incident angle is beyond *θ*_*c*_, the corresponding refractive angle can be predicted by the new law of refraction with *n* = 2 (see the iso-frequency contours in Fig. 4(c)). For example, when a plane wave is incident with *θ*_*i*_ = 30°, the calculated refractive angle is *θ*_*t*_ = −30°. To verify this scenario, we carry out numerical simulations as shown in Fig. 4(c), where we could find that the transmitted wave has a refractive angle *θ*_*t*_ = −30° for both the plane wave and Gaussian beam (see the insertion in Fig. 4(c)). The relationship between the wave transmittance and incident angle is revealed in Fig. 4(d). We can find that the transmittance is very high, except the angle around *θ*_*i*_ = 0°. That is because, when the angle is around *θ*_*t *_= 0°, the transmitted wave almost propagates along the surface of metallic grating, so that the transmittance of the wave is very low from far field observers.

#### (c) Metallic grating with *ξ* = 1.2*k*_0_

**Figure 5:**
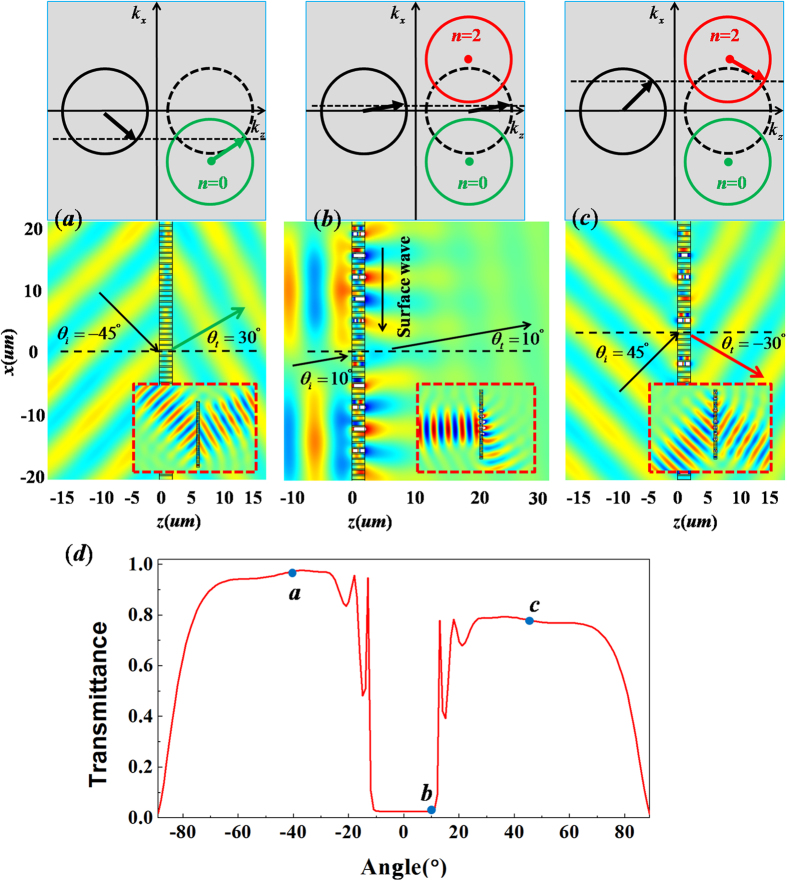
The case of metallic grating with *ξ* = 1.2*k*_0_. (**a**–**c**) are simulated magnetic field patterns for incident wave with different angles with *θ*_*i*_ = −45°, 10° and 45°, respectively. The upper parts are the corresponding iso-frequency contours, while the corresponding patterns of plane wave incident on metallic grating are placed in the nether parts, where the patterns for Gaussian beams bumping on the metallic grating with 6 supercells are inserted in bottom, which are marked by the red dashed frames. (**d**) is relationship between transmittance and incident angle. The points a, b and c in (**d**) denote the corresponding transmittance for the above cases of (**a**–**c**), respectively.

For the case of *ξ* = 1.2*k*_0_, the critical angle is shift to *θ*_*c*_ = −11.54°. As we have demonstrated that if the incident angle is within *θ*_*c*_, the refractive wave will obey the generalized Snell’s law (see the iso-frequency contours in Fig. 5(a)). The corresponding field patterns for a plane wave and a Gaussian beam with *θ*_*i*_ = −45°<*θ*_*c*_ are observed in Fig. 5(a), where the incident wave can pass through the metallic grating with a refractive angle *θ*_*i*_ = 30°, consistent with the predicted result. While for *θ*_*c*_ < *θ*_*i*_ <– *θ*_*c*_, it is noted from the iso-frequency contours in Fig. 5(b) that it is impossible to match the required tangential momentum with the *n* = 0 and *n* = 2 orders, *i.e*., the red and green circles. On simulations, for instance, considering that a plane wave with *θ*_*i*_ = 10° is incident on the grating structure. As shown in Fig. 5(b), we find that the wave is partly reflected, and some are confined along the interface between metallic grating and air, behaved as SSP. By observing the field pattern carefully, a very weak transmitted wave occurs with a refractive angle *θ*_*t*_ = 10°, which is labeled by a black arrow in Fig. 5(b). We also examine the result from such case, where a Gaussian beam is incident on the metallic grating with 6 supercells, as shown in the insertion of Fig. 5(b). For the incident wave with *θ*_*i*_ > −*θ*_*c*_ = 11.54°, it can match the required momentum with the *n* = 2 order from the iso-frequency contours in Fig. 5(c). The numerical simulation for the incident wave with *θ*_*t*_ = 45^°^ is shown in Fig. 5(c), where we find that the refractive angles of the transmitted wave are about *θ*_*t*_ = −30° for both plane wave and Gaussian beam, which is coincident with the result obtained by the new law of refraction. In Fig. 5(d), it displays the relationship between the transmittance of wave and the incident angle. We can find that the transmittance is high in the range of *θ*_*i*_ < *θ*_*c*_ and *θ*_*i*_ < −*θ*_*c*_, because the wave can pass through the metallic grating by following our new law of refraction. While for *θ*_*c*_ < *θ*_*i*_ < −*θ*_*c*_, most of incident wave is converted into SSP or is reflected strongly, giving rise to quite low transmittance for far field observers.

#### (d) Metallic grating with *ξ* = 2*k*_0_

**Figure 6:**
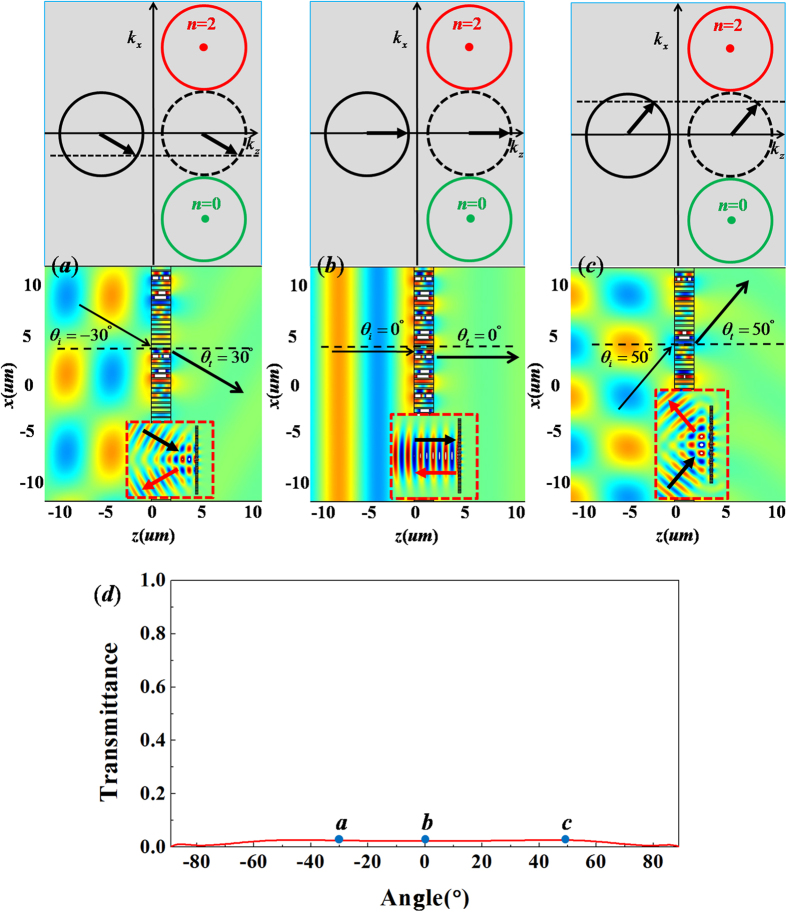
The case of metallic grating with *ξ* = 2*k*_0_. (**a**–**c**) are simulated magnetic field patterns for incident wave with different angles with *θ*_*i*_ = −30°, 0° and 50°, respectively. The upper parts are the corresponding iso-frequency contours, while the corresponding patterns of plane wave incident on metallic grating are placed in the nether parts, where the patterns for Gaussian beams bumping on the metallic grating with 6 supercells are inserted in bottom, which are marked by the red dashed frames. (**d**) is relationship between transmittance and incident angle. The points a, b and c in (**d**) denote the corresponding transmittance for the above cases of (**a**–**c**), respectively.

By observing three iso-frequency contours in Fig. (6), we can find that the incident wave with any incident angle could not couple to the green and red circles(*i.e., n* = 0 and *n* = 2). The incident wave is converted into SSP, leaking a weak transmitted wave with the refractive angle following the Snell’s law (see the simulations of plane wave in Fig. 6). In particular, from the simulations of Gaussian beams incident on the metallic grating with 12 supercells, we can find that the metallic grating behaves as a mirror, as the incident waves are reflected with a reflection angle equal to incident angle. Moreover, by observing the relationship between transmittance and the incident angle in Fig. 6(d), we can find that the transmittance is very low for all incident angles. Thus, such a metallic grating with *ξ* = 2*k*_0_ could well function as a mirror regardless of incident angle.

## Discussion

We have demonstrated that the metallic grating with discrete and gradient index materials can be used to manipulate light and steer its refracted directions. Such a structure is a reduced version of metasurface from transformation optics. It resembles some functionalities of metasurfaces from phase discontinuities. For example, when the incident angles are smaller than the critical angle, they follow the same generalized Snell’s laws. While for incident angles larger than the critical angle, the refracted waves in our structure are not just converted to surface waves, but also could be coupled to higher order grating modes, complying with new laws of refraction. Such a feature has been demonstrated in a recent acoustic metasurface design^21^, and some of the current work has been used to successfully explain the phenomena therein. Recently, the transmission and total reflection in controllable manner was demonstrated by another acoustic metasurface^23^, whose design is similar with our proposed configuration. However, as they only considered an extreme case of normal incident wave, some interesting physical phenomena were covered. We hope that our work will help to improve the understanding of such an acoustic metasurface.

Although several interesting optical phenomena have been illustrated in the proposed structure, which can be interpreted intuitively by the iso-frequency contours, yet the essential physics of grating diffraction of higher orders has not been revealed. Similarly, as metasurfaces also consists of periodic supercells, the grating diffraction of higher orders may appear for case of incident angles beyond the critical angle. Therefore, we hope that this work can arouse some research interest among related fields including metamaterials, plasmonics and grating diffraction, so that the issues we report in this work can be further explored. In addition, we expect that our proposed EM metasuface will be implemented in the coming future. In the current design, we choose free-space matched metamaterials in each unit cell of the grating, of which the physics behind is straightforward to understand, however at a price, not easy to implement at optical frequencies. In Supplementary Figure S1, we show that by keeping the refractive indexes of all the free-space matched metamaterials unchanged and changing them into normal dielectric materials, although there will be some reflection, the transmission (or refraction) still follows the same rule uncovered here.

## Additional Information

**How to cite this article**: Xu, Y. *et al.* Steering light by a sub-wavelength metallic grating from transformation optics. *Sci. Rep.* **5**, 12219; doi: 10.1038/srep12219 (2015).

## Supplementary Material

Supplementary Information
