# Supplementary material for: Steering light by a sub-wavelength metallic grating from transformation optics
Source: Sci Rep. 2015 Jul 17;5:12219. doi: 10.1038/srep12219 (PMC4505341; doi:10.1038/srep12219)
Supplement: Supplementary Information [file srep12219-s1.doc]

**Supplementary Information for:**

**Steering light by a sub-wavelength metallic grating from transformation optics**

Yadong Xu, Yangyang Fu and Huangyang Chen*

*College of Physics, Optoelectronics and Energy & Collaborative Innovation Center of Suzhou Nano Science and Technology, Soochow University, No.1 Shizi Street, Suzhou 215006, China.*

*[*chy@suda.edu.cn*](mailto:chy@suda.edu.cn)

**Supplementary Figures**


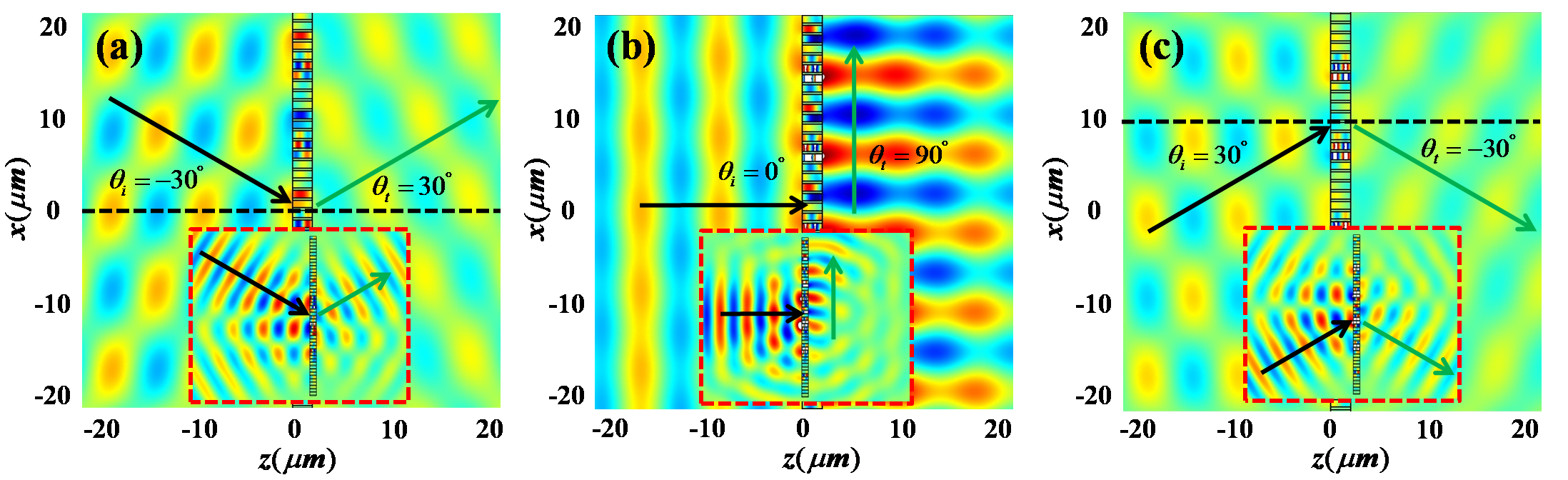


**Supplementary Figure S1. The case of metallic grating with , by changing all the free-space matched metamaterials ($\varepsilon=\mu=n$) in each unit cell in Figure 4 into normal dielectric materials ($\varepsilon=n^{2}$, $\mu=1$)**. (**a**), (**b**) and (**c**) are simulated magnetic field patterns for incident plane wave with different angles with $\theta_{i}=-30^{\circ}$, $0^{\circ}$ and $30^{\circ}$, respectively. The patterns for Gaussian beams bumping on the metallic grating with 6 supercells are inserted in the bottom, which are marked by the red dashed frames.
